# Supplementary material for: Breathing Control Exercises Delivered in a Group Setting for Patients with Chronic Obstructive Pulmonary Disease: A Randomized Controlled Trial
Source: Healthcare (Basel). 2023 Mar 17;11(6):877. doi: 10.3390/healthcare11060877 (PMC10048700; doi:10.3390/healthcare11060877)
Supplement: Supplementary file 1 [file healthcare-11-00877-s001.zip › healthcare-2238922-supplementary.pdf]

File S1

Breathing control exercises delivered in a group setting for patients with chronic obstructive pulmonary disease: a randomized controlled trial.

Breathing control exercises delivered in a group setting: frequency, intensity, time, and type.

There were 3-4 sessions per week (for a total of 20 sessions per program) of breathing control exercises in groups (5-6 participants per group), lasting 30 minutes per session. The latter sessions were provided by an occupational therapist. Breathing control exercises are defined as any breathing technique that can allow deeper inspiration or expiration, or otherwise alter the rate, pattern, or rhythm of respiration (Yun et al., 2021). Breathing techniques were incorporated during 15 different sets of body movements that had to be repeated 10 times each, with a recovery time of 10 seconds between each body movement. These movements were all open-chain upper-body and lower-body kinetic exercises, except for the sit-to-stand movement. Free weights (dumbbells, weighted balls, etc.) were given to the patients. The free weight loads of each exercise gradually increased over the sessions whenever the patient was able to perform 10 repetitions comfortably.

Description of body movements

Body movements were chosen according to their similarity with activities carried out in daily life (*e.g.,* passing one's hand in the back to wash, catching a product at height, bending down to tie one's laces, etc.). Each movement was associated with breathing control exercises. Patients were instructed to breathe in slowly and deeply during the concentric phase of each movement, then breathe out using pursed-lips breathing during the eccentric phase of the movement. Occupational therapists insisted on inspiration/expiration notions by encouraging patients during each movement.

Weighted ball	Resistive elastic band	Horizontal bar	Flexoring	Dumbbells
• Purple: no weight<br>• Yellow: 1 kg<br>• Orange: 1.5 kg<br>• Green: 2 kg<br>• Blue: 2.5 kg	• Black: Heavy Special<br>• Blue: Extra heavy<br>• Green: Heavy<br>• Red: Medium<br>• Yellow: light	• Yellow: no weight<br>• Grey: 1 kg<br>• Orange: 1.5 kg<br>• Red: 2 kg<br>• Blue: 2.5 kg	• Blue: normal<br>• Black: difficult	• Pink: 0.5 kg<br>• Yellow: 1 kg<br>• Red: 2 kg<br>• Grey: 2.5 kg<br>• Black: 3 kg

Examples of training with weighted balls:

1. Weighted ball held in two hands. Shoulder antepulsion (90 degrees) alternating between concentric and eccentric phases.
   - Inspiration (through the nose) during the concentric phase.
   - Expiration (through the mouth, lips pursed) during the eccentric phase.
2. Weighted ball held in two hands. Arm in front of (90 degrees) alternating rotation to the right and to the left.
   - Inspiration (deep breathing, through the nose), slow and conscious.

- Expiration (lips pursed), slow and conscious.

3. Weighted ball held in two hands. Shoulder antepulsion (180 degrees) alternating between concentric and eccentric phases.
   - Inspiration (through the nose) during the concentric phase.
   - Expiration (through the mouth, lips pursed) during the eccentric phase.
4. Feet firmly on the ground, weighted ball held between the knees. Hip adduction x10.
   - Inspiration (through the nose) during the concentric phase.
   - Expiration (through the mouth, lips pursed) during the eccentric phase.
5. Muscle relaxation exercise, without weight. Shoulders circumduction (for 30 s)
   - Inspiration (deep breathing, through the nose), slow and conscious.
   - Expiration (lips pursed), slow and conscious.
6. Muscle relaxation exercise, without weight. Head circumduction (for 30 s)
   - Inspiration (deep breathing, through the nose), slow and conscious.
   - Expiration (lips pursed), slow and conscious.
7. Weighted ball held in two hands. Bending to touch the ground and return to the initial position and then shoulder antepulsion (180 degrees).
   - Expiration on reaching the ground (lips pursed).
   - Inspiration when returning to the starting position (through the nose).
8. Weighted ball held in two hands. Simultaneous hip flexion/knee extension (alternating right and left) and shoulder antepulsion (90 degrees).
   - Inspiration (through the nose) during the concentric phase (during hip flexion and shoulder antepulsion).
   - Expiration (through the mouth, lips pursed) during the eccentric phase (during knee extension and returning to the starting position).
9. Weighted ball held in two hands. Knees bent, stand on tiptoes, with simultaneous shoulder antepulsion (90 degrees).
   - Inspiration (through the nose) during the concentric phase.
   - Expiration (through the mouth, lips pursed) during the eccentric phase.
10. Legs straight, heels anchored in the ground. Weighted ball held in two ankles. Hip flexion up to 90 degrees, and back to the starting position.
    - Inspiration (through the nose) during the concentric phase.
    - Expiration (through the mouth, lips pursed) during the eccentric phase.
11. Weighted ball held in two hands; arms extended at 90 degrees. Trunk rotation from left to right.
    - Inspiration (deep breathing, through the nose), slow and conscious.
    - Expiration (lips pursed), slow and conscious.
12. Weighted ball held in two hands; arms extended at 90 degrees. Shoulders and elbows flexion associated with left knee extension and left hip flexion (left).
    - Inspiration (through the nose) during the concentric phase.
    - Expiration (through the mouth, lips pursed) during the eccentric phase.
13. Weighted ball held in two hands; arms extended at 90 degrees. Shoulders and elbows flexion associated with right knee extension and right hip flexion (right).
    - Inspiration (through the nose) during the concentric phase.
    - Expiration (through the mouth, lips pursed) during the eccentric phase.
14. Get up (with or without armrests) with shoulder antepulsion (180 degrees).
    - Inspiration on rising (through the nose).
    - Expiration upon return on sitting (through the mouth, lips pursed).

Examples of training with a resistive elastic band:

1. Elastic held in hands, arms extended at 90 degrees. Open arms and return to the initial position.
   - Inspiration (through the nose) during the concentric phase.
   - Expiration (through the mouth, lips pursed) during the eccentric phase.
2. Elastic in the back, sit on one end. Hold the other end of the elastic then elbow extension to reach 180 degrees of antepulsion.
   - Inspiration (through the nose) during the concentric phase.
   - Expiration (through the mouth, lips pursed) during the eccentric phase.
3. Muscle relaxation exercise. Head circumduction.
   - Inspiration (deep breathing, through the nose), slow and conscious.
   - Expiration (lips pursed), slow and conscious.
4. Muscle relaxation exercise. Shoulders circumduction.
   - Inspiration (deep breathing, through the nose), slow and conscious.
   - Expiration (lips pursed), slow and conscious.
5. Elastic held in hands. Arms in front (90 degrees), then perform a horizontal arms abduction, maintaining respiratory pause for 3 seconds, and then return to the initial position.
   - Inspiration (through the nose) during the concentric phase.
   - Expiration (through the mouth, lips pursed) during the eccentric phase.
6. Bend to touch the floor, straighten up, raise arms up to 180 degrees, and open the chest by stretching the elastic band behind the head.
   - Expiration on reaching the ground (lips pursed).
   - Inspiration when returning to the starting position (through the nose).
7. Hip flexion combined with knee extension simultaneous with horizontal arm abduction, and return to the initial position.
   - Inspiration (through the nose) during the concentric phase.
   - Expiration (through the mouth, lips pursed) during the eccentric phase.
8. Alternating tiptoes simultaneously with opening arms (horizontal shoulder abduction) in front and returning to the initial position.
   - Inspiration (through the nose) during the concentric phase.
   - Expiration (through the mouth, lips pursed) during the eccentric phase.
9. Left hand on the left hip holding the end of the elastic band, and the other hand on the other end, stretch the elastic band to reach 180 degrees of antepulsion position.
   - Inspiration (through the nose) during the concentric phase.
   - Expiration (through the mouth, lips pursed) during the eccentric phase.
10. Right hand on the right hip holding the end of the elastic band, and the other hand on the other end, stretch the elastic band to reach 180 degrees of antepulsion position.
    - Inspiration (through the nose) during the concentric phase.
    - Expiration (through the mouth, lips pursed) during the eccentric phase.
11. Shoulder antepulsion (180 degrees) and abduction to stretch the elastic.
    - Inspiration (through the nose) during the concentric phase.
    - Expiration (through the mouth, lips pursed) during the eccentric phase.
12. Get up (with or without armrests) with shoulder antepulsion (180 degrees).
    - Inspiration on rising (through the nose).
    - Expiration upon return on sitting (through the mouth, lips pursed).
